# Isolation and characterization of spontaneously immortalized B‐lymphocyte lines from HIV‐infected patients with and without non‐Hodgkin's Lymphoma

**DOI:** 10.1002/cam4.2508

**Published:** 2019-09-20

**Authors:** Ke Zhuang, Yongxi Zhang, Li Zhou, Xiaoying Qi, Xiqiu Xu, Fengzhen Meng, Zhigao Xu, Jinbiao liu, Liang Shao, Huan Liu, Hang Liu, Jun Fang, Di Deng, Jianhong Peng, Fuling Zhou, Li Liu, Hongbin Tang, Yong Xiong, Wenzhe Ho, Deying Guo, Hengning Ke, Xien Gui

**Affiliations:** ^1^ ABSL‐III Laboratory at the Center for Animal Experiment State Key Laboratory of Virology Wuhan University Wuhan China; ^2^ Department of Infectious Diseases Zhongnan Hospital of Wuhan University Wuhan China; ^3^ Basic Medical College of Wuhan University Wuhan China; ^4^ Department of Pathology Zhongnan Hospital of Wuhan University Wuhan China; ^5^ Department of Hematology Zhongnan Hospital of Wuhan University Wuhan China; ^6^ AIDS Institute and Department of Microbiology Li Ka Shing Faculty of Medicine The University of Hong Kong Hong Kong SAR China; ^7^ Department of Radiation Oncology Zhongnan Hospital of Wuhan University Wuhan China; ^8^ Department of Clinical Laboratory Medicine Zhongnan Hospital of Wuhan University Wuhan China; ^9^ School of Basic Medicine (Shenzhen) Sun Yat‐sen University Guangdong China

**Keywords:** B‐lymphocyte, HIV infection, immortalized, NHL, spontaneous

## Abstract

Isolation of viable circulating tumor cells (CTC) holds the promise for improving screening, early diagnosis, and personalized treatment of lymphoma. In this study, we isolated and characterized spontaneously immortalized B‐lymphocyte (SIBC) lines from HIV‐infected patients with and without Non‐Hodgkin's Lymphoma (AIDS‐NHL). A total of 22 SIBC lines was isolated from peripheral blood mononuclear cells (PBMC) of HIV‐infected patients with (n = 40) and without (n = 77) clinically detectable NHL, but not from healthy individuals (n = 34). Of these, 8 SIBC lines named HIV‐SIBC were generated from HIV‐infected patients without AIDS‐NHL (10%, 8/77), while 14 SIBCs named AIDS‐NHL‐SIBC were from 13 of the AIDS‐NHL patients (32.5%, 13/40). Among the 14 AIDS‐NHL‐SIBCs, 12 were derived from AIDS‐NHL patients with poor prognoses (survival time less than 1 year). SIBCs displayed markers typical of memory B cells (CD3^‐^CD20^+^CD27^+^) with EBV infection. Moreover, AIDS‐NHL‐SIBCs were representative of CTC as evidenced by monoclonal Ig gene rearrangement, abnormal chromosomal karyotype, and the formation of xenograft tumors, while HIV‐SIBCs generated harbored some features of tumor cells, none had the capacity of xenograft tumor formation, suggesting HIV‐SIBC present the precursor of CTC. These results indicate that SIBCs is associated with poor prognosis in AIDS‐NHL patients and can be isolated from HIV‐infected patients with NHL and without NHL. This findings point to the need for further molecular characterization and functional studies of SIBCs, which may prove the value of SIBCs in the diagnosis, prognoses, and screening for NHL among HIV‐infected patients.

## Graphical abstract

Spontaneously immortalized B‐lymphocyte (SIBC) lines were generated by culturing PBMCs from either HIV‐infected patient with or without AIDS‐NHL. SIBCs are representative of the CTCs and associated with poor prognosis in AIDS‐NHL patients, while SIBCs from HIV‐infected patients without NHL could be a precursor of CTC.

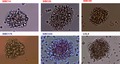

## 1 INTRODUCTION

The morbidity and mortality of Non‐Hodgkin's Lymphoma (NHL) rank seventh and ninth among malignant tumors, respectively. The incidence of NHL is approximately 60‐100 times higher in the HIV‐infected population as compared with the general population.^1^, ^2^ In our clinic at Wuhan, China, AIDS‐NHL is the most common malignant disease among HIV‐infected patients, with early onset and rapid disease progression.^3^ Currently, tissue biopsy and imaging remain the main methods used for the diagnosis and prognosis of cancer, with liquid biopsies gaining extensive attention in the past decades. Liquid biopsies in blood include circulating tumor cells (CTCs) and cell‐free nucleic acids (cfNAs). Compared to cfNAs, the isolation of viable CTCs is technically more demanding. However, whole viable tumor cells are of greater value than assays at the nucleic level, due to CTCs available for the functional analyses.^4^, ^5^, ^6^ Unfortunately, culturing/propagating of CTCs is still challenging.^7^ Despite the vast literature on CTC research, only approximately 50 publications reported culturing CTCs. In addition, these CTCs were mostly cultured for short periods (3‐14 days),^8^, ^9^ with only a few studies successfully establishing immortalized CTC lines.^8^, ^10^, ^11^ Moreover, the rare investigations focused on analyzing the correlation between CTC lines and clinical manifestations. As we know, early screening is crucial for the prevention of cancers in high‐risk populations. Several studies showed that CTCs meeting the criteria for “tumor‐cells” were detected in high‐risk individuals,^12^, ^13^ which suggested that tumor cells might already be present in the benign inflammatory lesions. It is regrettable that these cells were not isolated for subsequent culture, analyses, and functional characterization.

Here, we isolated spontaneously immortalized B‐lymphocytes (SIBCs) from the blood of HIV‐infected patients with and without NHL and then characterized their biological features, as well as analyzed whether establishment of SIBC lines would be a valuable indicator of clinical significance in AIDS‐NHL.

## 2 MATERIALS AND METHODS

### 2.1 Patients and blood collection

HIV‐infected patients diagnosed with (n = 40) and without (n = 77) AIDS‐NHL between January 2015 and November 2018 in Zhongnan Hospital were enrolled in this study. The diagnosis of lymphoma was based on WHO classification.^14^ Medical records of the patients were collected, including age, gender, diagnosis, and chemotherapy. Patients were followed up for clinical information. For controls, 34 healthy subjects without symptoms of recent or ongoing fever and without enlarged lymph nodes were recruited for this study. The study was approved by the Ethics Committee of Zhongnan Hospital of Wuhan University. Informed consent was obtained from all patients. All experiments were performed in accordance with the ethical standards established in the Declaration of Helsinki.

Blood (5‐10 mL) was collected in EDTA‐treated tubes. Peripheral blood mononuclear cells (PBMC) were separated by Ficoll‐hypaque density gradient centrifugation at 800× *g* for 30 minutes. Then, the mononuclear layer was collected and washed twice in PBS then cultured in complete RPMI 1640 Medium containing fetal bovine serum (FBS). The detailed procedure was described as follows.

### 2.2 Establishment of SIBC lines

PBMCs isolated from patients and healthy controls were used to establish cell lines in vitro. PBMCs were divided into two aliquots, for the establishment of lymphoblastoid cell lines (LCLs) and SIBCs, respectively. SIBC lines were established by culturing 1 × 10^7^ PBMCs in RPMI 1640 supplemented with 20% FBS, 1 × Glutamine, 1 × HEPES and 1 × Penicillin/Streptomycin (Life Technologies) at 37°C in 5% CO_2_ for the first 3 weeks, then switched to 10% FBS RPMI 1640 for at least 8 weeks, and the cultures were fed with a half‐change of medium weekly. LCLs were established by infecting PBMCs (1 × 10^7^) with EBV overnight, and EBV‐transformed cells were then cultured in 20% FBS RPMI 1640 with the presence of cyclosporine A at a concentration of 0.1g/ml in order to avoid any coincidental T‐cell activation in vitro within the first 3 weeks. Cells were then fed weekly with a half‐change of 10% FBS medium without cyclosporine A. The EBV for LCL transformation was prepared and stocked as a culture supernatant of an EBV‐producing marmoset cell line, B95‐8. Using microscopy, all cultures were observed weekly for cell growth. Once growth began, cell numbers gradually increased until some of the cells could be frozen in liquid nitrogen and some of the cells could be used for this study. Cells were then passaged once weekly and maintained for at least 6 months.

### 2.3 Phenotype analysis

Phenotype analysis was performed by flow cytometry and Immunofluorescence. Using a panel of anti‐human antibodies used for the clinical pathological diagnosis (Table S1), phenotype analysis for SIBCs and LCLs was carried out as described.^15^, ^16^ Cells were first washed with PBS and then stained with different anti‐human monoclonal antibodies or isotype control antibodies for 30 minutes. The isotype control antibodies (BD Biosciences) used in this study includes PE‐Cy™7 Mouse IgG1 κ Isotype Control (Clone MOPC‐21, cat.557872), APC Mouse IgG1 κ Isotype Control (Clone MOPC‐21, cat.554681), and APC‐H7 Mouse IgG2b, κ Isotype Control (Clone 27‐35, cat.560183). Ten thousand events were collected on a FACSCanto flow cytometer (BD), and FACS analyses were performed using FlowJo software (v7.6.1; TreeStar). As for Immunofluorescence labeling for SIBCs, cells were fixed with 4% polyformaldehyde in PBS and then permeabilized with PBS containing 0.5% Triton X‐100 for 20 minutes at room temperature. After washing with PBS and blocking with goat serum, cells were generally incubated at 4°C overnight with primary antibodies from different species (mouse or rabbit) and followed by Cy3‐labeled goat anti‐mouse (red). The cell nuclei were stained with 4′,6‐diamidino‐2‐phenylindole (DAPI) for 5 minutes in the dark and visualized by a fluorescence microscope (Olympus BX53 Microscope). Omission of the primary antibody served as a negative control.

### 2.4 Molecular B‐cell clonality assessment

Detection of immunoglobulin heavy chain (IGH) and light chain (kappa, IGK; IG lambda, IGL) gene rearrangements was performed using the standardized BIOMED‐2 multiplex PCR assays according to the manufacturer's protocols (Gene Scan: Lymphoma Gene Rearrangements Assay, Yuanqi Biomedical). Genomic DNA (200 ng) was amplified using AmpliTaq Gold DNA Polymerase. PCR conditions involved an initial denaturation step of 95°C for 7 minutes followed by 40 cycles of 95°C for 45 s, 60°C for 45 s, 72°C for 90 s, and a final step of 72°C for 10 minutes. The quality of isolated DNA was assessed. Amplified PCR products were analyzed for size distribution on an ABI 3500 DxGenetic Analyzer (Applied Biosystems) according to standard procedures. Analysis and interpretation of the amplified gene products were performed using Gene mapper software (Applied Biosystems).

### 2.5 G‐banding karyotype analysis

The SIBC cell suspension was placed in 25 cm^2^ culture flasks (Corning) for Cytogenetic examination. 5 μg/mL colcemid (Gibco‐Life Technology) was added into the SIBCs suspension for 2 hours to arrest cell mitosis. Cells were treated with 0.075 mol/L KCl and then fixated with 3:1 methanol:glacial acetic acid (Sinopharm Chemical Reagent). Cell solution was dropped onto a slide glass and air‐dried. Then, cells were treated with 0.005% trypsin for 7 minutes and stained with 6% Giemsa stain solution for 3.5 minutes. The results were processed with Applied Spectral Imaging. The number of chromosomes was analyzed in 30~50 cells, and a detailed karyotype was analyzed in 10~11 cells. The definitions were followed the International System for Human Cytogenetic Nomenclature (ISCN) 2013.

### 2.6 Mouse xenograft assays

The animal use protocol was approved by the Institutional Animal Care and Use Committee (IACUC) of Wuhan University. Six‐week‐old male NOD‐SCID mice (Weitonglihua) were allowed to acclimatize for 1 week under specific pathogen‐free (SPF) conditions in the animal facility of the Center for Animal Experiment. Mice were used in eight groups of three to five mice each and anesthetized with 2,2,2‐tribromoethanol, then 5~10 × 10^6^ (5 000 000~10 000 000) cells in 100 µl of PBS were subcutaneously injected into mice at the thigh (right and left) or right shoulder. Tumors were monitored twice‐weekly for tumor onset. The tumor volume was calculated according to the formula (length × width^2^)/2. Mice with tumors greater or equal to 500 mm^3^ were sacrificed, and the tumor was sectioned and stained for their histological analysis.

### 2.7 Immunohistochemistry analysis of xenograft

The subcutaneous xenografts formed in NOD‐SCID mice were cut in 4‐μm sections and analyzed with the following primary antibodies (Table S1). Immunohistochemistry was carried out as described.^11^ Briefly, sections were deparaffinized and rehydrated before heat‐mediated epitope retrieval was performed. Endogenous peroxidase activity was quenched by 10‐minute incubation in 3% hydrogen peroxide. After incubation with blocking solution consisting of PBS containing 2% bovine serum albumin (BSA) for 30 minute, sections were incubated with primary antibodies (CD20, CD5, CD10, BCL‐2, BCL‐6, Mum‐1, c‐Myc, Cyclin D1, and Ki67) for 1 hour. Biotinylated secondary antibody was then incubated for 20 minutes, followed by streptavidin‐horseradish peroxidase (HRP) for 10 minutes at room temperature and visualized by 3,3‐diaminobenzidine (DAB), resulting in a brown‐colored precipitate at the antigen site.

### 2.8 Measurement of EBV viral load

Genomic DNA was isolated from cells using the QIAamp DNA mini kit (QiagenInc) according to the manufacturer's instructions. DNA was diluted with 50 μL nuclease‐free water (Life Technologies) and DNA concentration was calculated. Quantitative PCR assay was performed using a real‐time PCR assay with Bio‐Rad MyIQTM 2. Concentrations of EBV DNA were measured using primers flanking the BamHI‐W region of the EBV genome. The sequence of the forward and reverse primers of the BamHI W region was 5′‐CCCAACACTCCACCACACC‐3′ and 5′‐TCTTAGGAGCTGTCCGAGGG‐3′, respectively. A dual‐labeled TaqMan probe (5′‐FAM‐CACACACTACACACACCCACCCGTCTC‐BHQ‐1‐3′) served as a probe. The Namalwa (ATCC® CRL‐1432TM) DNA was used to create a six‐point 10‐fold serial dilution series, ranging from 10^6^ copies/μL to 10^1^ copies/μL, that was used to obtain the standard curve.

### 2.9 Phylogenetic analysis of full‐length LMP1

The full‐length LMP1 gene was amplified with nest‐PCR. The first round of PCR was completed by using the following primers: LMP1‐F1 (B95‐8 coordinate 168012‐168032; 5′‐TAGAATATGAATGTGGCTTTT‐3′) and LMP1‐R1 (B95‐8 coordinate 169716‐169737; 5′‐CAAACACACGCTTTCTACTTCC‐3′). The second round of PCR was performed with 1 μL of template from the first round reaction and the following primers: LMP1‐F2 (B95‐8 coordinate 168057‐168077, 5′‐AGGGAGTGTGTGCCAGTTAAG‐3′) and LMP1‐R2 (B95‐8 coordinate 169584‐169603; 5′‐ACACTCGCACAGCCCACACC‐3′). The PCR reaction was set up in a reaction volume of 50 μl using Primer STAR GC polymerase (TaKaRa), and then reactions were carried out in a GeneAmp PCR system (Applied Biosystems). PCR product length was approximately 1500 KB. Primers LMP1‐F2, LMP1‐R2 and LMP1‐S3 (5′‐GGAGGGAGTCATCGTGGTGGT‐3′) were used for sequencing.

Full‐length LMP1 DNA sequences were aligned using the CLUSTAL W multiple alignment, using LMP1 from the following five known strains as references: B95‐8 Prototype (accession no. V01555.1), Raji (accession no. NC_007605), AG876 (accession no. DQ279927.1), Alaskan (accession no. AY337725.1), China1 (accession no. AY337723.1), and China2 (accession no. AY337724.1). Neighbor‐joining phylogenetic trees were generated by MEGA version 5.0.

### 2.10 Statistical analysis

Clinical and laboratory data of patients in AIDS‐NHL group, HIV+ group and control group were compared. Comparisons were carried out using t test using version 6.0 of Prism (Graph‐Pad Software). All statistical tests were used in the two‐sided variant, and *P* values less than .05 were considered statistically significant.

## 3 RESULTS

### 3.1 Patient characteristics

During the period 2015‐2018, a total number of 40 AIDS‐NHL cases and 77 HIV‐infected cases (HIV+) without clinical indications of NHL were enrolled in this study. Thirty‐four healthy cases were included as a control group. Table 1 summarizes the characteristics of the AIDS‐NHL and HIV+ patients. Male patients accounted for a higher proportion in both groups, representing 85% (34/40) of the AIDS‐NHL and 78% (60/77) of the HIV+ patients. At the initial time of sampling, patients in the AIDS‐NHL group had higher HIV‐RNA plasma viral load than the HIV+ group (*P* = .07). Among the 40 AIDS‐NHL patients, the two most common types of lymphomas are DLBCL (45%) and BL with/without ALL (28%), which together accounted for 73% of all cases. The most common extranodal site of AIDS‐NHL is the gastrointestinal tract, followed by the central nervous system and liver. Most patients were chemotherapy‐naïve at the time of enrollment, with chemotherapy initiated as soon as the diagnosis of AIDS‐NHL was made.

**Table 1:** Patient characteristic at baseline

Characteristic	AIDS‐NHL	HIV+	*P*‐values^a^
Overall	40	77	
Age, median (range)	46 (14‐66)	42 (5‐64)	.5
<20	1 (2%)	2 (3%)	
20‐39	11 (28%)	25 (32%)	
40‐59	24 (60%)	47 (61%)	
60 and above	4 (10%)	3 (4%)	
Gender			
Female	6 (15%)	17 (22%)	
Male	34 (85%)	60 (78%)	.3
CD4^+^ count (cells/µL), median (range)^b^	251 (4‐591)	301 (9‐1444)	.1
<150	9 (23%)	22 (28%)	
150‐350	13 (32%)	14 (18%)	
350‐750	15 (38%)	32 (42%)	
>750	0 (0%)	3 (4%)	
Unknown	3	6	
Plasma HIV RNA (copies/mL), median (range)^b^	142,000 (0‐2,160,000)	55 000 (0‐890 000)	.07
Undetectable	19 (48%)	41 (53%)	
Detectable	18 (45%)	30 (39%)	
Unknown	3	6	
CART^b^			
Naïve	21 (52%)	27 (35%)	.08
NRTI + NNRTI	19 (48%)	50 (65%)	
B‐Cell NHL histotype		None	
DLBCL	18 (45%)		
BL w/wo ALL	11 (28%)		
B lymphoma	9 (23%)		
FL	1 (2%)		
CD	1 (2%)		
Tumor site		None	
Nodal	12 (30%)		
Extranodal	11 (28%)		
Nodal + Extranodal	17 (42%)		
Survival time			
<1 y	22 (55%)	4(5%)	<.0001^c^
>1 y	13 (32%)	54 (70%)	
Exclude	5	19	

Abbreviations: ALL, acute lymphoblastic leukemia; B lymphoma, Unclassified B‐NHL; BL, Burkitt lymphoma; cART, combination antiretroviral therapy; CD, castleman's disease; DLBCL, diffuse large B‐cell lymphoma; Exclude, excluding patients who were lost to follow‐up, less than 1‐year follow‐up, or died from AIDS‐unrelated causes.; FL, follicular lymphoma; None, not any.

a The *t* test was used in the statistical analysis of the two groups (AIDS‐NHL & HIV+).

b At the initial time of sampling.

c The value was significantly different.

Excluding patients who were lost to follow‐up, less than 1‐year follow‐up, or died from disease‐unrelated causes (eg, suicide), 35 AIDS‐NHL and 58 HIV‐infected patients were reached for follow‐up assessment of their clinical information (Table 1). The patients were divided into two groups of survival time <1 or >1 year. The results showed that survival time <1 year was significantly higher in the AIDS‐NHL (55%) than the HIV+ group (5%, *P* < .0001), with a median survival time of 164 days for the AIDS‐NHL patients (1~363 days). Thirteen patients with >1 year survival time were currently still alive except one.

### 3.2 Establishment of SIBCs and LCLs

**Figure 1:**
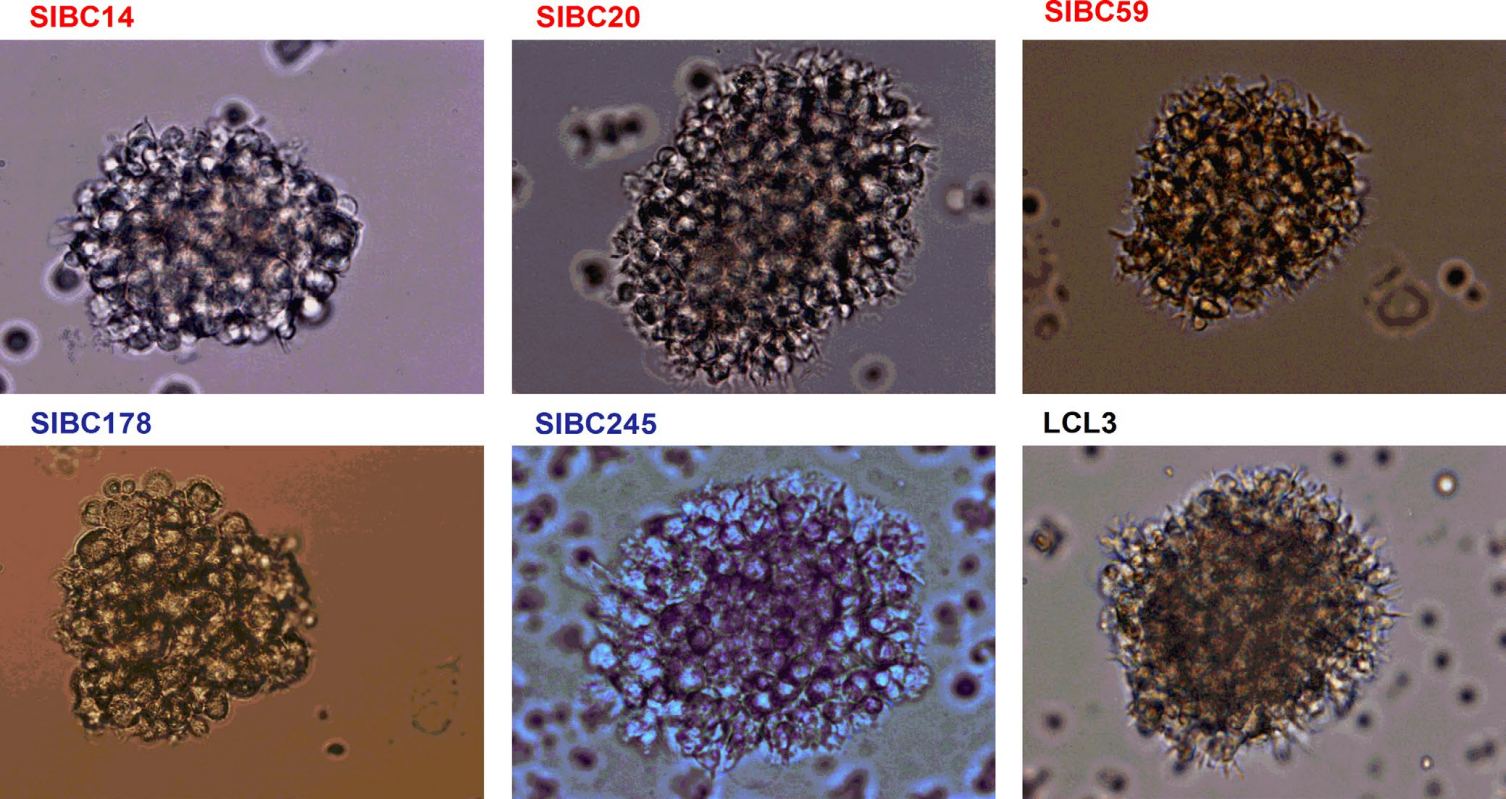
Representative images of ex vivo expansion of SIBC lines. SIBC14, 20, and 59 were generated from AIDS‐NHL patients, in which a pairs of SIBC lines (SIBC14 and SIBC20) were established from blood samples drawn at the period of prechemotherapy and interval chemotherapy in patient ZNA003; SIBC178 and 245 were derived from HIV+ patients; LCL3 was established by EBV (B95‐8 strain) transformation of PBMCs from a health individual. Scale bar: 100 μm

Lymphoblastoid cell lines (LCLs) were generated with the classical protocol for establishing LCLs.^17^ The cell morphology of the LCLs presented a rapid growth and formation of cell clumps in suspension observed after 1‐2 weeks of culture. PBMCs from patients and healthy individuals were cultured to generate SIBC lines in vitro. Fourteen AIDS‐NHL‐SIBC lines (35%) were generated from the 40 AIDS‐NHL patients, in which two are “sister” lines (SIBC14, 20) derived from patient (ZNA003) before and during chemotherapy. In comparison, only 8 HIV‐SIBC lines were generated from 77 HIV‐infected patients, with none established from the 34 healthy controls (Table 2). SIBC lines usually started to grow after 3‐7 weeks in culture (mean of 4.5 weeks). Similar to LCLs, SIBCs initially formed several small round shape clumps in suspension which then expanded to larger numbers within approximately 10 weeks (Figure 1). Once established, SIBC lines could be sustained in vitro for 6~24 months. By analyzing various clinical factors, we found only the survival time of AIDS‐NHL patients was associated with SIBC line generation. SIBC lines were derived from 12 (~50%) of the 22 AIDS‐NHL patients with survival time <1 year as compared to only 2 (~15%) from the 13 patients with >1 year survival time (*P* = .003).

**Table 2:** Cinical timepoint of SIBC culture

Clinical diagnosis	Patients cases, N	SIBC, N (%)^a^	Clinical characteristic		Cases, N	SIBC, N (%)^a^	*P*‐values
AIDS‐NHL	40	14 (35%)^b^	Clinical stage	I‐II	15	6 (40)	0.5
				III‐IV	25	8 (32)	
			B symptoms	Yes	17	7 (41)	0.4
				No	23	7 (30)	
			IPI	0‐1	5	2 (40)	
				2‐3	21	9 (43)	0.2
				4‐5	14	3 (21)	
			Tumor site	Nodal	12	4 (33)	0.5
				Extranodal w/wo Nodal	28	10 (36)	
			Plasma LDH level	Normal	16	5 (31)	0.6
				High	24	9 (38)	
			Survival time^c^	<1 y	22	12 (54)	0.003^d^
				>1 y	13	2 (15)	
HIV+	77	8 (10%)^b^	Survival time^c^	<1 y	4	1 (25)	0.5
				>1 y	54	7 (13)	
Healthy	34	0			32	0	
Total	151	22					

a The number (percentage) of established SIBC lines.

b The value between AIDS‐NHL and HIV+ groups was significantly different, *P* = .001.

c Excluding patients who were lost to follow‐up, less than 1‐year follow‐up, or died from AIDS‐unrelated causes.

d The value between <1 y and >1 y groups was significantly different.

### 3.3 Cell origin and Immunophenotype of SIBC cells

**Figure 2:**
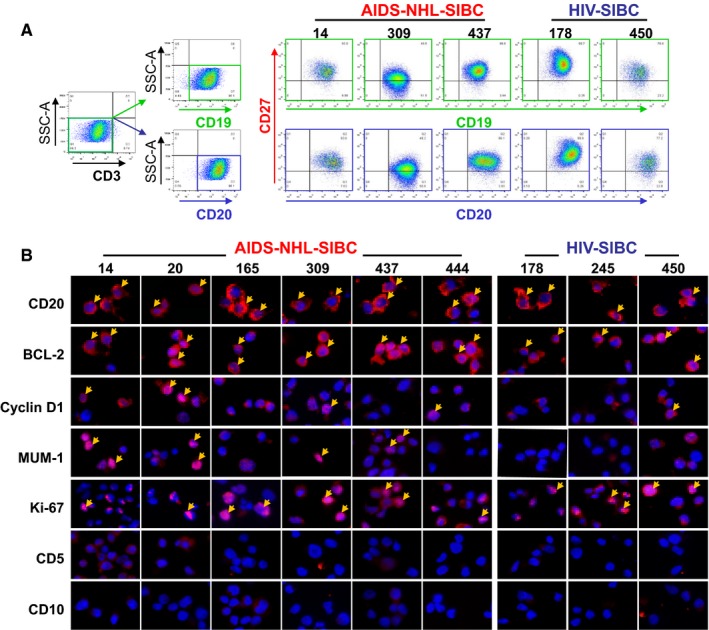
Representative flow cytometric analysis and immunofluorescence staining of SIBC lines. A, Phenotype analysis of B‐lymphocytes of SIBC lines by flow cytometry with anti‐CD3, anti‐CD19, anti‐CD20, and anti‐CD27 antibodies. Immunophenotypes of cell lines were determined as negative for CD3 and positive for CD20 and CD27. CD20^+^CD27^+^ memory B cells were a predominant population in all SIBC lines. Number indicates percentage of cells in the marked gates. B, AIDS‐NHL‐SIBC and HIV‐SIBC lines were strongly positive for CD20, BCL‐2, and Ki67 (shown in red), while the expression of Mum‐1 and Cyclin D1 protein was different among SIBC cells. The yellow arrow indicates representative positive expression. Scale bar: 20 μm

The origin and immunophenotype of the SIBCs were analyzed using B‐cell surface markers. High expression of CD19 or CD20 was observed on >95% of the cells. CD19^+^/CD20^+^CD27^+^ memory B cells predominated in all SIBC lines, accounting for ~90% (Table S2). Only two SIBC lines (AIDS‐NHL‐SIBC309 and HIV‐SIBC450) have a lower proportion of CD19^+^/CD20^+^CD27^+^ memory B cells, accounting for ~48% in SIBC309 and ~77% in SIBC450 (Figure 2A). The predominance of memory B cells in the SIBC lines is similar to that observed in the EBV‐transformed LCL lines, with 93% of the cells being CD19^+^/CD20^+^CD27^+^.

To further characterize the SIBCs, staining with a panel of antibodies used for the clinical pathological diagnosis of lymphoma tissue was performed. Results are presented in Figure 2B, showing a high degree of similarity in the immunophenotype of AIDS‐NHL‐SIBCs and HIV‐SIBCs. Both SIBC lines strongly expressed CD20 and BCL‐2 but negatively for CD5 and CD10. Ki67 also stained strongly in the majority of SIBCs, while differences in expression of Mum‐1 and Cyclin D1 protein were noted among the SIBC lines.

### 3.4 Ig gene rearrangements of SIBC cells

**Figure 3:**
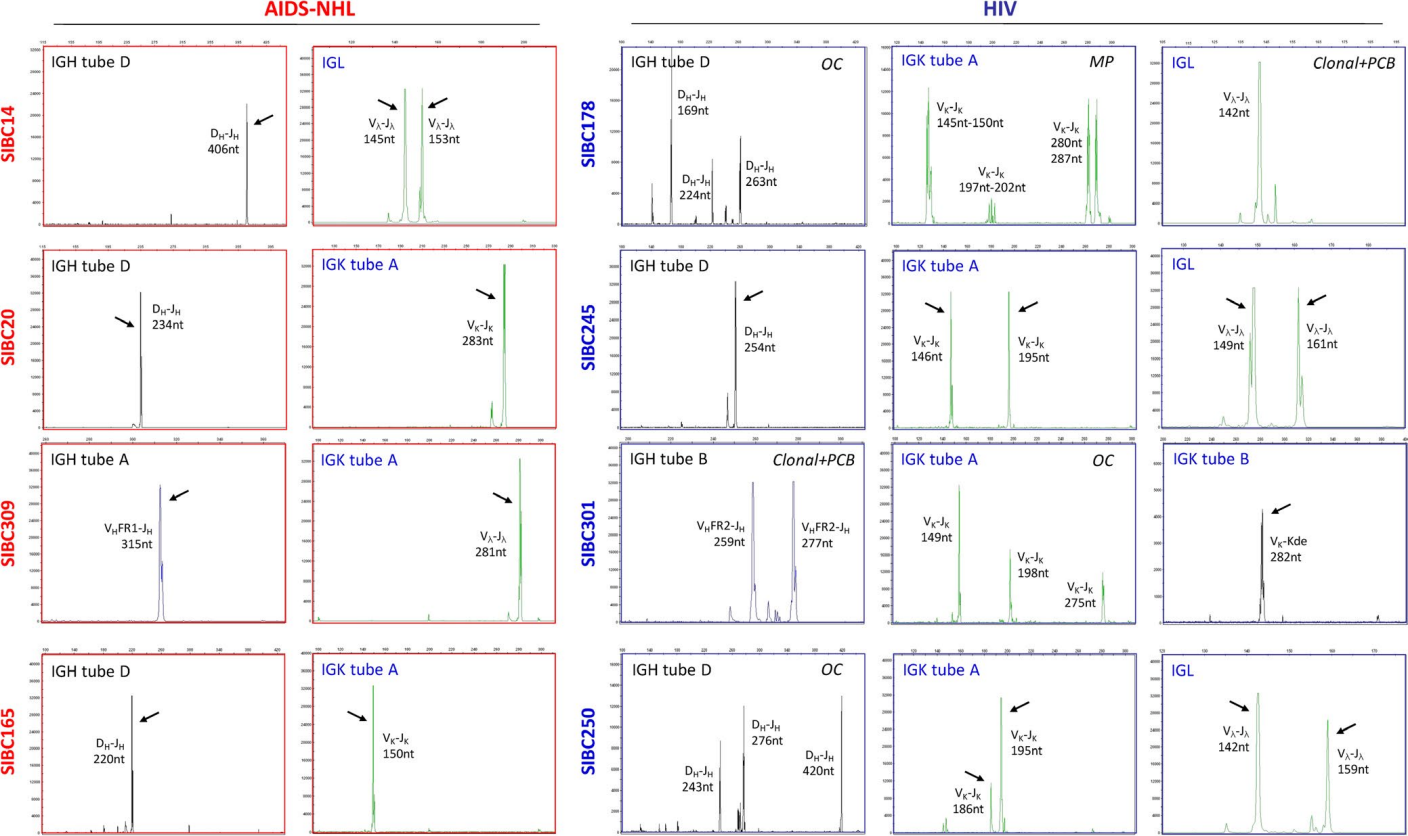
Representative molecular B‐cell clonality assessment of SIBC lines by Ig gene rearrangements. Monoclonal rearrangement identified by unequivocal monoallelic or biallelic clonal gene rearrangement peak(s) were detected in all AIDS‐SIBCs and HIV‐SIBC245 (indicated by arrows). Polyclonal arrangement identified by oligoclonality (OC), multiple products (MP) or clonal in polyclonal background (Clonal+pcb) were detected in HIV‐SIBC lines. IGH, Ig heavy chain; IGK, Ig kappa light chain; IGL, Ig lambda light chain

In order to demonstrate that SIBCs are the malignant B‐lymphocytes, an IGH, IGK, and IGL gene rearrangement assay was performed on 12 SIBC lines, including seven AIDS‐NHL‐SIBC and five HIV‐SIBC lines. All AIDS‐NHL‐SIBCs showed monoclonal rearrangements of the Ig gene (IGH and/or IGL), indicative of the malignant origin of SIBCs (Figure 3; Table S3). IGH/IGK expression was the most frequent pattern detected in AIDS‐NHL‐SIBCs, except SIBC14 and 20 which presented IGH rearrangement with dual expression of IGK and IGL light chain.

In contrast, five of the seven HIV‐SIBC lines characterized (178, 245, 250, 251, and 301) showed diverse and complex rearrangements of the Ig gene, including monoclonal rearrangement, oligoclonal rearrangement (OC), monoclonal rearrangement in the background of polyclonal rearrangement (Clonal+PCB), and multiple products (MP). HIV‐SIBCs 245 and 251 showed monoclonal rearrangements, SIBCs 250 and 301 exhibited monoclonal and oligoclonal rearrangements, while all four patterns of Ig gene rearrangements were detected in SIBC178. Similar to AIDS‐NHL‐SIBC14 and 20, three HIV‐SIBCs (178, 245, and 250) have the dual presence of IGK and IGL light chain (Figure 3; Table S3).

### 3.5 Chromosomal karyotype of SIBC cells

To examine the chromosomal characteristics of SIBCs, 30~50 metaphase SIBCs were counted for chromosome numbers and 10~11 were analyzed for chromosome structure (Table 3). Most SIBCs presented more or less aneuploidy (2n±m) and tetraploid/near tetraploidy (T/NT, 4n±m, 81~103 chromosomes), with four SIBCs (165, 437, 444, and 245) showing higher frequencies of aneuploidy or T/NT. SIBC165 had marked aneuploid with trisomy 7 (47, XX, +7) that accounted for 80% (24/30), while 37% (11/30) of SIBC444 presented a 45, XY karyotype due to random loss of one chromosome. Thirty‐three percent (18/54) of SIBC437 and 20% (6/30) of SIBC245 were characterized with the T/NT karyotype (86‐92, XX or XY).

**Table 3:** Results of cytogenetic examinations of SIBC lines

Diagnosis	Patient ID	Clinical diagnosis of NHL	SIBCs	Number of chromosomes				Structural of chromosomes	
				N	2n/2n ± m	3n/3n ± m	4n/4n ± m	N	Karyotype
AIDS‐NHL	ZNA003	DBLCL	14	32	46[26]/44[1]	0	89[2], 90[3]	10	44‐46,XY,del(3)(p21)[1],‐8[1],‐9[1], chtg(21)(q21)[1] /46,XY[9]
	ZNA003	DBLCL	20	28	46[26]	0	90[2]	10	46,XY[10]
	ZNA005	BL/ALL	165	30	46[2]/43[2], 44[1], **47[24]**	0	91[1]	10	43‐47, XX, **dup(1)(q25‐q42)[10]**, −4[1],**+7[9]**, −8[1], chrb(9)(P22)[1],‐12[1], **t(8;14)(q24;q32) [9],**add(14)(q32)[1], −17[1], ‐X[1]/46, XX[1]
	ZNA013	BL	309	35	46[31]/45[2], 47[1]	0	91[1]	10	45‐46,XY,chtg(2)(p11)[1],chtg(3)(q13)[1],dic(3;5)(q25;q23)[1],del(3)(q27)[1],chtg(4)(q27)[1],t(4;8)(p14;q21)[2], t(5;17)(q31;p13)[1],‐5[1],chtg(5)(q11)[1],del(5)(q31)[1],chtg(5)(q15)[1],t(6;22)(q23;p13)[1], chth(6)(p21)[1], chtg(8)(q23)[1],t(9,22)(q34;q11)[1],del(10)(p13)[1],chtg(10)(q21)[1],chtg(11)(q14)[1],chtg(13)(q12)[1],del(13)(q32)[1],del(17)(q24)[1],add(22)(P11)[1],‐22[2],i(22q)[1],+M[1],+M1[1],+M2[1]/46,XY[9]
	ZNA024	DLBCL	437	54	46[32]/43[2]	60[1], 76[1]	**92[11]**/86[1], 89[2], **91[4]**	10	43‐47,XX, chtg(2)(q36))[1], +8[1], −8[1], chtg(12)(q13)[1], −13[1],‐13[1], chtg(15)(q22)[1], add(18)(p11)[1]/ 91, XX, idem(or sdl) x2, −9[1], −19[1]/ 46, XX[6]
	ZNA027	BL	444	30	46[12]/41[1], 44[2], **45[11]**, 47[1]	0	92[2]/90[1]	10	44‐46,XY,‐19[1],‐22[1],‐12[1],‐8[1],‐11[1],‐18[1],t(Y;6)(p25;p11)[1]/46,XY[4]
	ZNA028	BL	448	30	46[22]/43[2], 45[3], 47[1]	0	92[1]/91[1]	11	45‐46, XY, **t(8;14)(q24;q32)[9]**,chtg(10)(p13)[1], −19[1]/46, XX[10]
	ZNA031	B lymphoma	455	30	46[22]/41[1], 42[2], 44[2], 45[2], 47[1]	0	0	10	41‐46,XY,‐71[1],‐8[1],‐9[1],‐12[1],‐13[1],‐15[1],‐16[1],‐21[1],‐22[1]/46,XY[7]
HIV+	ZNB007	None	178	30	46[27]/43[1], 45[1],	0	89[1]	10	43‐46,XY,‐8[1],‐12[1],‐20[1],‐Y[1]/46,XY[8]
	ZNB039		245	30	46[15]/42[3], 45[3], 47[1], 50[1]	60[1]	**92[5]/**88[1]	10	45‐46,XY,chtg(4)(q22)[1],chtg(8)(p21)[1],‐12[2],t(8;21)(p23;q22)[1],chtg(22)(q12)[1]/46,XX[8]
	ZNB034		250	30	46[18]/43[2], 44[1], 45[2], 48[1]	71[1]	85[2], 90[1], 93[1], 99[1]	10	45‐48,XY,chtg(1)(q32)[1],chtg(2)(q31)[1], chtg(5)(q21)[1],‐11[1],‐13[1], del(17)(p11)[1],‐19[1],‐22[1],del(22)(q13)[1], ‐Y[1],‐Y[1],+m,+r /46,XY[6]
	ZNB068		450	30	46[24]/42[1], 43[2], 44[1], 45[1]	0	86[1]	10	45‐46,t(1;15)(q25;q26)[1],‐17[1]/46,XX[9]

2n/2n±m: diploid/aneuploidy.

3n/3n±m: triploid/hypertriploid or hypotetraploid.

4n/4n±m: tetraploid/near tetraploid.

N: The number of chromosomes was analyzed.

Numbers in square brackets [xx] refer to the absolute numbers of chromosome.

Higher frequency of chromosome numbers abnormalities and clonal structural abnormalities were highlighted in bold font.

**Figure 4:**
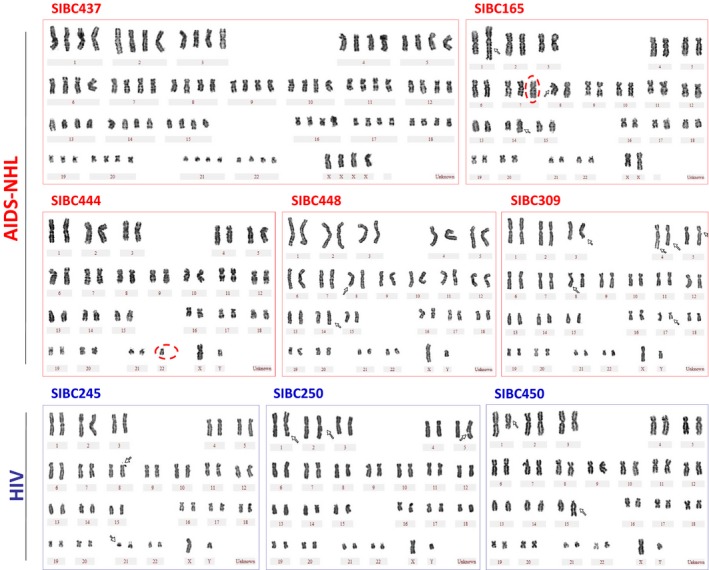
Representative results of G‐banded karyotype of SIBC lines. Various structural and/or numerical aberrations were detected in both AIDS‐NHL‐SIBC and HIV‐SIBC lines. SIBC437 presented a tetraploid karyotype; SIBC165 showed marked aneuploid with trisomy 7 (47,XX,+7), as well as dup(1)(q25‐q42) and t(8;14)(q24;q32); SIBC444 had random loss of chromosome 22, resulting in 45,XY aneuploid; SIBC448 also harbored a clonal abnormality t(8;14)(q24;q32); the cytogenetic diagnosis of SIBC309 was 46,XY,del(3)(q27),chtg(4)(q27),t(4;8)(p14;q21),chtg(5)(q11),del(17) (q24); SIBC245 exhibited chtg(8)(p21) and t(8;21)(p23;q22); SIBC250 mainly showed the aberration of chtg, with chtg(1)(q32),chtg(2)(q31),chtg(5)(q21); and SIBC450 had a t(1;15)(q25;q26). Chtg, chromatidgap; del, deletion; dup, duplication; t, translocation; Arrowheads indicate the structural abnormality; red‐dotted circle show the add or loss of chromosome

Moreover, structural abnormalities including terminal deletion, insertion, translocation, and chromatid gap were detected in the majority of SIBCs, with only SIBC165 and SIBC448 exhibited clonal chromosomal structural abnormalities. Ninety percent of SIBC165 and SIBC448, which were derived from patients ZNA005 and ZNA028 respectively with BL harbored the translocation t(8;14)(q24;q32), which is a characteristic marker for Burkitt's lymphoma (Figure 4; Table 3). Also, 100% of SIBC165 carried a duplication on 1q25‐q42 chromosome dup(1)(q25‐q42). The results suggested that chromosomal abnormalities existed in the majority of SIBC lines derived from both AIDS‐NHL and HIV+ patients, with more severe abnormalities detected in AIDS‐NHL‐SIBC lines.

### 3.6 Growth of SIBC cells as xenografts in NOD‐SCID mice

**Figure 5:**
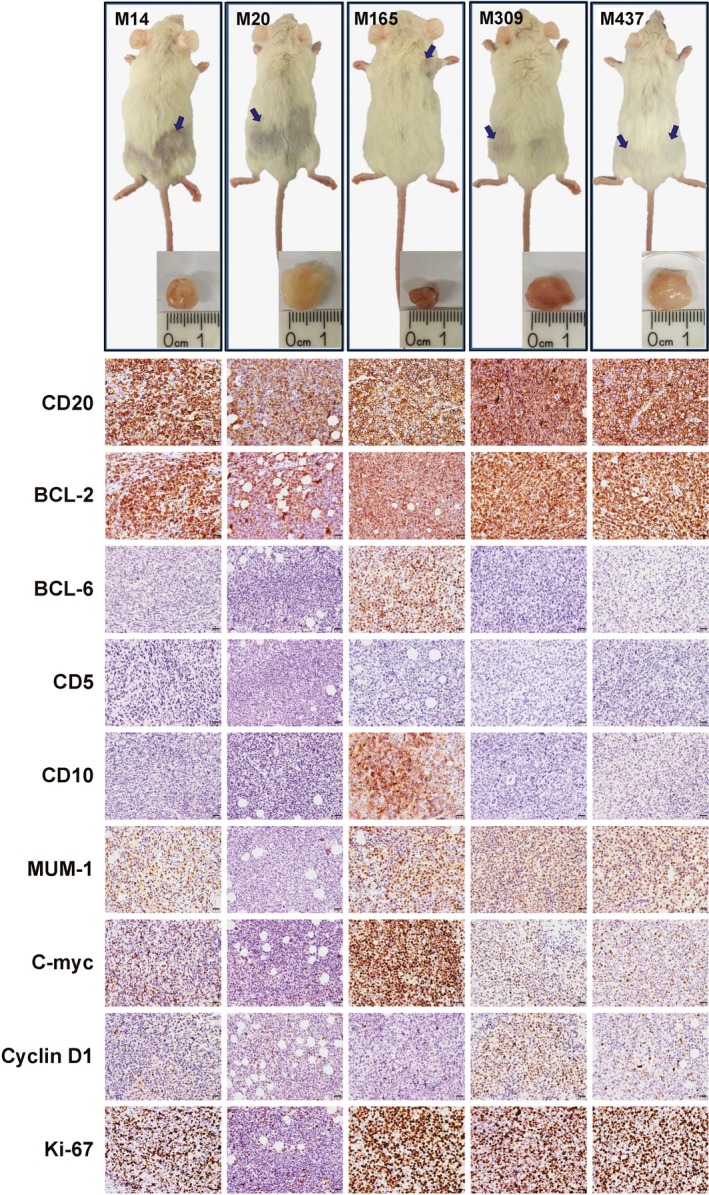
Tumorigenicity of SIBCs in NOD‐SCID mice. (upper panels) the formation of tumor xenografts (blue arrows) could be observed in NOD‐SCID mouse after implantation of 5 ~ 10 × 10^6^ cultured AIDS‐NHL‐SIBCs subcutaneously. (lower panels) the Immunohistochemical analysis of matched SIBC‐derived mouse xenografts showed cellular staining with hematoxylin (blue) and immunohistochemical staining for positive expression (brown). Scale bar, 20 mm

To further characterize the tumorigenicity of SIBCs, five AIDS‐NHL‐SIBCs and three HIV‐SIBCs were subcutaneously inoculated into NOD‐SCID mice. All five AIDS‐NHL‐SIBCs (14, 20, 165, 309, and 437) generated tumors but not the three HIV‐SIBCs (250, 245, and 178). AIDS‐NHL‐SIBC derived xenografts (M14, M20, M165, M309, and M437) shared similar but not identical immunohistochemical features with the corresponding SIBC cells (Figure 5; Table S4). The staining of the xenografts showed intense CD20 expression in all xenografts tissue, with the expression rate exceeded 95% in each samples, but negative for CD5 staining. With the exception of M20, all tumors expressed high levels of BCL‐2 and Ki67 proteins (≥80%), with varying expression levels of MUM‐1, c‐Myc, and Cyclin D1 observed in different xenografts. Although xenograft M14 and M20 were extracted from the mice inoculated with the pair of "sister" cells SIBC14 and SIBC20, respectively, the expression levels of positive staining in M20 is significantly lower than M14. The results demonstrated AIDS‐NHL‐SIBCs but not HIV‐SIBCs have the capacity of tumor formation.

### 3.7 EBV DNA level and phylogenetic analysis of the LMP1 DNA sequences

**Figure 6:**
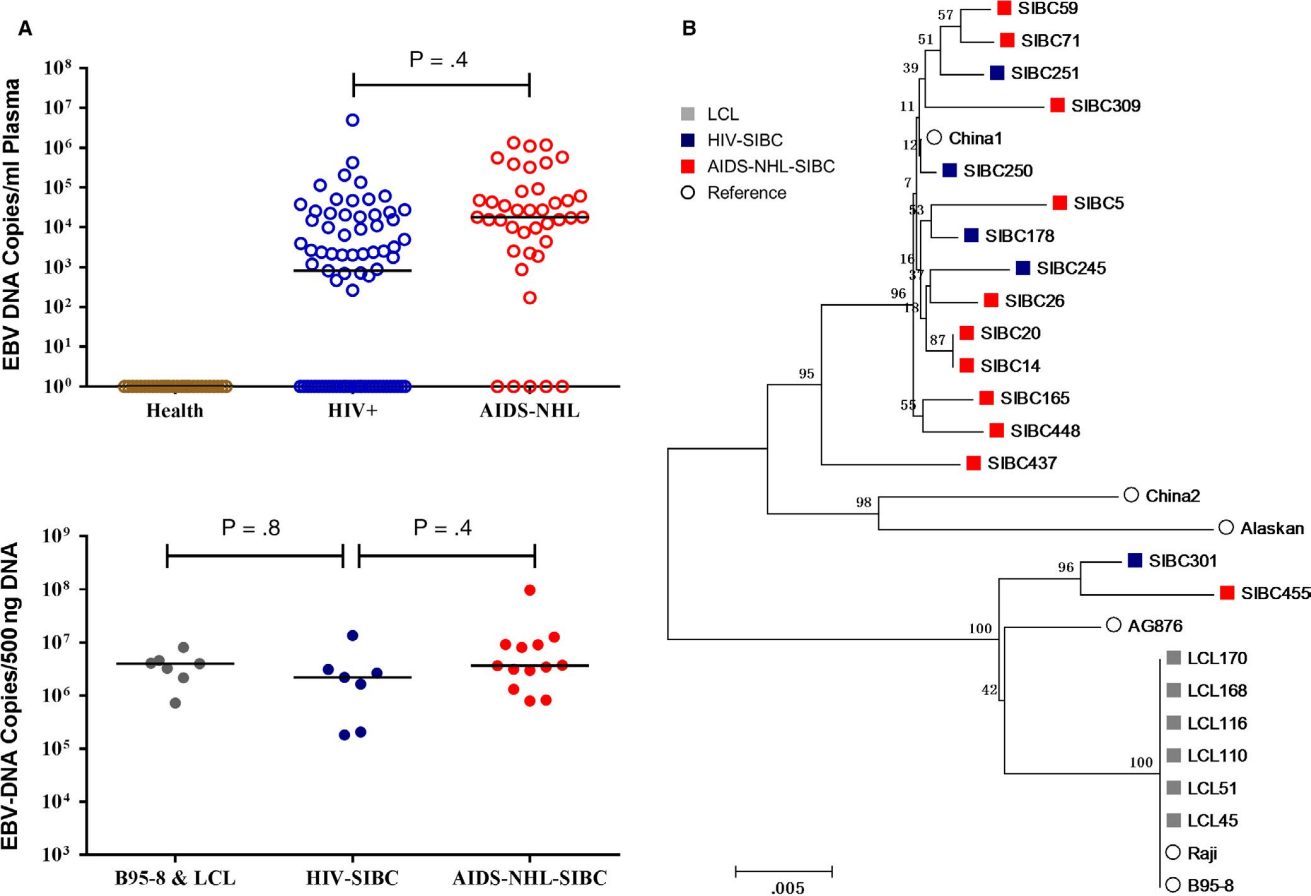
EBV analysis in SIBC. A, EBV DNA level in plasma of healthy individuals, HIV+ and AIDS‐NHL patients, as well as cellular EBV DNA level in B95‐8&LCL, HIV‐SIBC and AIDS‐NHL‐SIBC lines, detecting DNA using primers located in BamHI W regions. B, A phylogenetic tree of the LMP1 sequences from SIBC and LCL lines. A neighbor‐joining tree of the LMP1 gene is shown, with bootstrap values of 70% indicated at the appropriate nodes. Genetic distances representing the number of nucleotide substitutions per site between LMP1 sequences (0.005) are indicated at the bottom of each tree

The overall EBV‐DNA levels detected in the plasma of AIDS‐NHL patients was higher than that in the HIV+ patient group (median, 1.8 × 10^4^ and 8.1 × 10^2^ copies/mL, *P* = .4), and was undetectable in healthy individuals (Figure 6A). In addition, all SIBCs had a EBV‐DNA level comparable to B95‐8 and LCLs, with the median level of 3.0 × 10^6^ copies/500 ng DNA (1.8 × 10^5^~9.7 × 10^7^), but there was no significant difference in EBV‐DNA levels between HIV‐SIBC and AIDS‐NHL‐SIBC lines (Figure 6A). A neighbor‐joining phylogenetic tree was constructed with LMP1 sequences of SIBCs, LCLs and references. Phylogenetic analyses showed that LMP1 sequences from LCLs clustering in a lineage were highly similar and closely related to the reference B95‐8 and Raji strains. In contrast, the majority of SIBCs appeared to cluster with the China 1 strain but not the China 2 strain, which were both isolated from the tumor tissue of a Chinese patient with nasopharyngeal carcinoma (Figure 6B), while two SIBCs (301 and 455) clustered with AG876 strains derived from Ghanaian Burkitt's lymphoma. EBV‐DNA levels and EBV‐LMP1 sequence analysis showed EBV infection is a common event in HIV‐infected patients and significant homologies with EBV from SIBC lines and tumor tissue of Chinese patients.

### 3.8 Nucleotide sequence accession numbers

The nucleotide sequence data of LMP1 from 16 representative SIBCs reported in the present paper are available in the GenBank database under accession numbers MK637833‐MK637848.

## 4 DISCUSSION

It is still challenging to establish CTC lines from patients with tumor even with overt distant metastases. This study provides, for the first time, the experimental proof that SIBC lines can not only be isolated from AIDS‐NHL patients (32.5%) but also from HIV+ individuals without clinically detectable NHL (10%). Moreover, we observed the association between the cell line establishment and clinical prognosis is highly significant. Of the 14 SIBC lines generated from AIDS‐NHL patients, 12 were derived from 11 refractory NHL patients with survival time <1 year (median 268 days, range 39‐363 days). In this regard, CTCs detected in the blood of patients with metastatic cancer^18^, ^19^ have been reported to be predictors of therapeutic response^20^, ^21^, ^22^ and patient prognosis.^18^, ^23^, ^24^ Survival of CTCs requires the ability to overcome mechanical shear forces present in the bloodstream and attacks by intrinsic immunological responses.^25^ It is tempting to speculate that some amount of malignant lymphocytes already existed in circulation in AIDS‐NHL patients with poor prognoses, but few or not in patients with relatively good prognoses. Nonetheless, we failed to establish SIBC lines from another half of AIDS‐NHL patients with survival time <1 year. We found the median survival time in SIBC‐negative patients with survival time <1 year was 106 (2‐294) days, which was shorter than SIBC‐positive patient's 268 (39‐363) days, suggesting that it is much more difficult to generate SIBCs in near‐terminal stage patients. More studies urges comprehensive approach are needed to elucidate the reason.

Our findings supported AIDS‐NHL‐SIBCs are CTCs with several malignant B‐lymphocyte features, including monoclonal Ig gene rearrangement, Chromosome abnormality and xenografts. Monoclonal Ig gene rearrangement of AIDS‐NHL‐SIBCs mainly involved the IGH and/or IGK gene. Interestingly, the “sister” cell lines SIBC14/SIBC20 presented dual IGK/LGL light‐chain rearrangements, with reported in a few cases of B‐cell leukemia/lymphomas.^26^, ^27^ Moreover, we found the postchemotherapy‐derived SIBC20 showed increased IGK rearrangement compared with prechemotherapy‐derived SIBC14, consistent with reports that Ig gene rearrangements might change during chemotherapy.^28^, ^29^ Most AIDS‐NHL‐SIBCs exhibited numerical and structural chromosomal abnormalities, with high frequencies of aneuploidy or T/NT detected in AIDS‐NHL‐SIBC165, 437, and 444, and with the t(8;14)(q24;q32) translocation found in 80%‐90% of Burkitt's lymphoma cases being present in 90% of AIDS‐NHL‐SIBC165 and 448. One hundred percent SIBC165 carried duplications on 1q25‐q42 chromosome that were common genetic changes in acute leukemia and lymphoma. It is well documented that aneuploidy is present in approximately 86% of solid tumors and 72% of hematopoietic cancers,^30^, ^31^ and often reflects chromosomal instability (CIN) characterized by an increase in the rate of gain or loss of fragments or entire chromosomes. CIN and aneuploidy cause phenotypic variation and increase tumor heterogeneity, which is a reliable marker of high malignancy, poor prognosis, and drug resistance.^32^, ^33^, ^34^ However, “sister” lines SIBC14/SIBC20 showed less or no aberrations. We speculated the conventional G‐banding karyotype analysis did not effectively identify complex or subtle chromosomal abnormalities.^35^, ^36^ Further analysis, such as whole‐genome profiling may be required to reveal additional abnormal changes related to tumorigenesis. Lastly, we proved the tumor formation capacity of AIDS‐NHL‐SIBCs in mice. These data demonstrate that AIDS‐NHL‐SIBCs belong to CTCs.

HIV infection is a risk factor for NHL. Unexpectedly, we got SIBCs in 10% (8/77) of HIV+ patients. HIV‐SIBCs exhibited immunophenotypic characteristics similar to those of AIDS‐NHL‐SIBCs, but had more complex Ig gene rearrangement patterns and slighter aberration of chromosome than AIDS‐NHL‐SIBCs, with all HIV‐SIBCs harboring more and less aneuploidy and T/NT. It has been documented that early premalignant stages of several tumors are featured with increased levels of T/NT cells.^37^, ^38^ A study showed that tetraploid cells accumulate in the premalignant condition of Barrett's esophagus, which is interdependent of the loss of the p53 tumor suppressor but precedes the appearance of aneuploidy.^39^ However, HIV‐SIBCs failed to form xenograft in mice. We speculated HIV‐SIBCs might be a type of tumor precursor cells in blood of HIV‐infected patients. Therefore, our findings are consistent with others in detecting CTCs among patients at high risk for cancer.^12^, ^13^ More importantly, we successfully obtained viable HIV‐SIBCs and performed detailed phenotypic and functional assays. A long‐term follow‐up on SIBC‐positive HIV+ patients are required to monitor the development of complications. Moreover, a deep genomic and molecular biology research on SIBC lines is required to uncover accurate and general biomarkers.

All SIBC lines had an obviously high level of cellular EBV DNA, suggesting that the EBV infection and activation might contribute to the formation of immortalized B cells. EBV usually establishes lifelong latent infection in the memory B cell pool. The SIBC lines of this study presented CD19^+^/CD20^+^CD27^+^ memory B cells as a main population, while two AIDS‐NHL‐SIBC309 and HIV‐SIBC450 harbored a subpopulation of CD19^+^/CD20^+^CD27^−^ naïve B cells, suggesting that SIBCs were derived from either a CD27^‐^ germinal center (GC) or CD27^+^ post‐GC B cell.

In summary, we established and characterized SIBC lines from HIV‐infected patients with and without NHL. The advantages and significance of SIBC lines are as follows: (a) blood samples can be obtained easily and frequently without surgical operation; (b) SIBCs might have implications in clinical prognoses; (c) SIBCs might be a potential biomarker for NHL monitoring in high‐risk population. Nevertheless, our study had certain limitations, such as relatively small sample size, the difficulty to establish SIBC lines in near‐terminal stage AIDS‐NHL patients, lack of long‐term follow‐up of NHL high‐risk populations, and a longer period of ex vivo culture to generate SIBC lines. Further in‐depth studies on SIBC lines could be of great value for illuminating the Non‐Hodgkin lymphomagenesis in HIV‐infected patients and developing personalized medicine approaches.

## CONFLICT OF INTEREST

The authors declare no conflict of interest.

## AUTHOR CONTRIBUTIONS

All authors read and approved the manuscript. KZ and XG conceived and designed the experiments. KZ, YZ, LZ, XQ, XX, FM, HL, JF, HL, and JL conducted the experiments. YZ, LS, DD, JP, and FZ collected the clinical samples. KZ, YZ, LL, YX, ZX, HT, WH, DG, HK, and XG analyzed the data. KZ and XG wrote the manuscript.

## Data Availability

The data used to support the findings of this study are available from the corresponding author upon request.
