# Supplementary material for: Isolation and characterization of spontaneously immortalized B‐lymphocyte lines from HIV‐infected patients with and without non‐Hodgkin's Lymphoma
Source: Cancer Med. 2019 Sep 20;8(15):6741–55. doi: 10.1002/cam4.2508 (PMC6825990; doi:10.1002/cam4.2508)
Supplement: Supplementary file 1 [file CAM4-8-6741-s001.docx]

**Supplementary Tables**

**Supplementary Table 1：List of antibodies for phenotype analysis**

**Supplementary table 2. Flow analysis of SIBCs**

**Supplementary table 3: Ig rearrangment analysis of SIBCs**

**Supplementary table 4: comparison of immunophenotype of xenograft and corresponding SIBCs.**
